# Role of mitochondria transmission in ischemic stroke: Friend or foe?

**DOI:** 10.1016/j.redox.2025.103868

**Published:** 2025-09-20

**Authors:** Lan Yang, Xuan Wei, Zi Liao, Bei Chen, Guang Zeng, Zhigang Mei

**Affiliations:** a Key Laboratory of Hunan Province for Integrated Traditional Chinese and Western Medicine on Prevention and Treatment of Cardio-Cerebral Diseases, School of Integrated Traditional Chinese and Western Medicine, Hunan University of Chinese Medicine, Changsha, Hunan, 410208, China; b Academy of Chinese Medical Sciences, Hunan University of Chinese Medicine, Changsha, Hunan, 410208, China

**Keywords:** Ischemic stroke, Oxidative stress, Dysfunctional mitochondrial fragments, Functional mitochondria transfer, Neuroinflammation

## Abstract

Ischemic stroke ranks as the second leading cause of mortality and the third disability worldwide. Disruption of energy metabolism and subsequent inflammation driven by oxidative stress constitute significant barriers to functional recovery. Proper distribution and function preservation of mitochondria are essential for maintaining energy homeostasis and modulating the inflammatory response during cerebral ischemia and reperfusion injury. Accumulating evidence indicates that both dysfunctional mitochondrial fragments and functional mitochondria undergo intracellular and intercellular transmission, significantly influencing stroke outcomes. The review details two contrasting mitochondrial processes in ischemic stroke: the release of dysfunctional mitochondrial fragments into the cytoplasm or extracellular space and the entry of functional mitochondria into damaged cells, which plays a dual role: friend or foe. The release of dysfunctional fragments activates downstream pattern recognition receptors, including the cyclic GMP-AMP synthase–stimulator of interferon genes pathway, NLR family pyrin domain containing 3/absent in melanoma 2 inflammasome, and Toll-like receptors, triggering inflammatory cascades within the neurovascular unit and initiating cell death pathways contributing to cerebral injury. In contrast, the transfer of functional mitochondria plays a protective role by attenuating oxidative stress, preserving mitochondrial quality control, restoring neuronal energy metabolism, inhibiting apoptosis, and maintaining blood-brain barrier integrity. Therapeutic approaches that inhibit the release of dysfunctional mitochondrial fragments, enhance functional mitochondria transfer, or apply mitochondrial transplantation offer significant potential for improving outcomes in ischemic stroke.

## Highlights

• Cellular energy crisis drives mitochondrial transmission in ischemic stroke (IS).
• Mitochondrial transmission has a dual role in IS pathogenesis.
• ROS-induced mitochondrial fragmentation worsens neuroinflammation via PRRs signaling.
• Functional mitochondrial transfer rescues injured neurons by energy supply.
• Targeting mitochondria transmission is a promising therapeutic strategy for IS.

## Graphical abstract

The role of dysfunctional mitochondrial fragments and functional mitochondria in ischemic stroke.

**Figure:**
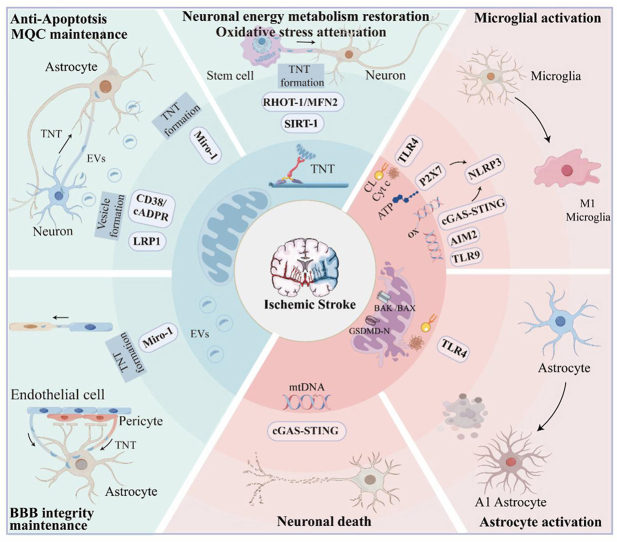

## 1 Introduction

Ischemic stroke ranks as the second leading cause of mortality and the third major contributor to global disability. Pathologically, it is characterized by an acute collapse of energy metabolism resulting from cerebral blood flow interruption [1,2]. Following arterial occlusion by thrombi or emboli, the neurovascular unit (NVU) rapidly depletes adenosine triphosphate (ATP) due to hypoxia and aglycemia, leading to disrupted ion gradients, membrane depolarization, and excitotoxic calcium overload [3]. Although reperfusion partially restores cerebral perfusion, it paradoxically aggravates mitochondrial oxidative stress through excessive production of reactive oxygen species (ROS), causing structural and functional damage to mitochondrial DNA (mtDNA), membrane proteins, and lipid components [4]. These pathological processes disrupt energy metabolism and amplify neuroinflammatory responses, thereby accelerating the progression of ischemic injury. Mitochondria, the central regulators of cellular energy metabolism, play a dynamic and dualistic role in ischemic stroke by releasing dysfunctional mitochondrial fragments and mediating intercellular transfer of functional mitochondria, thereby influencing injury progression and recovery [5].

The mitochondrial membrane system forms the core structural basis for cellular energy metabolism, consisting of the outer mitochondrial membrane (OMM), inner mitochondrial membrane (IMM), and intermembrane space (IMS), which collectively establish dual physical and functional barriers [6]. Under physiological conditions, the OMM facilitates selective metabolite exchange through voltage-dependent anion channels (VDACs), while the IMM maintains cristae architecture to support the function of electron transport chain (ETC) complexes in establishing a proton gradient. This gradient is then utilized by ATP synthase for ATP synthesis, and adenine nucleotide translocase (ANT) mediates the transmembrane transport of adenosine diphosphate (ADP) and ATP [6,7]. Pathological stress induces mitochondrial matrix swelling and cristae disintegration, leading to a collapse of the mitochondrial membrane potential (ΔΨm) and impairment of oxidative phosphorylation, ultimately producing dysfunctional mitochondria [8]. Dysfunctional mitochondria release various damage-associated molecular patterns (DAMPs), including mtDNA, Cytochrome *c* (Cyt *c*), cardiolipin (CL), and ATP, through three major permeabilization pathways: opening of the mitochondrial permeability transition pore (mPTP), Bcl-2-associated X protein (BAX)/Bcl-2 homologous antagonist/killer (BAK) mediated OMM permeabilization, and gasdermin D (GSDMD) formed membrane pores [9]. These mitochondrial DAMPs activate cytoplasmic pattern recognition receptors (PRRs), such as the cyclic GMP-AMP synthase–stimulator of interferon genes (cGAS-STING) axis, NLR family pyrin domain containing 3 (NLRP3)/absent in melanoma 2 **(**AIM2) inflammasomes, and Toll-like receptors (TLRs), which amplify neuroinflammation and contribute to cell death [10]. Furthermore, extracellular dysfunctional mitochondrial fragments act as stress signals recognized by surrounding cells [11], which subsequently transfer functional mitochondria to injured cells through a process known as functional mitochondria transfer [12]. Functional mitochondria, defined by intact outer and inner membranes, preserved cristae, matrix, and intermembrane space, generate ATP efficiently via oxidative phosphorylation to support cellular activity. During the progression of ischemic stroke, intercellular functional mitochondria transfer has emerged as a critical compensatory mechanism for tissue repair [13]. Growing evidence indicates that damaged cells receive functional mitochondria from adjacent cells through tunneling nanotubes (TNTs) and extracellular vesicles (EVs) [14] This specialized mode of intercellular communication not only replenishes ATP in energy depleted cells but also contributes to functional recovery by preserving mitochondrial quality, inhibiting apoptotic pathways, and maintaining the structural integrity of the blood-brain barrier (BBB) [12,15].

The present review provides a comprehensive analysis of the mechanisms underlying mitochondrial fragments release from dysfunctional mitochondria within the NVU, focusing on the pathways and therapeutic potential of functional mitochondria transfer in ischemic stroke. The goal is to elucidate the intricate interplay between mitochondrial content release and intercellular functional mitochondria transfer, thereby providing new insights into their potential as therapeutic targets for ischemic stroke.

## 2 Ischemic stroke pathophysiology

Ischemic stroke is a prevalent cerebrovascular disorder with multiple risk factors, including hypertension, smoking, diabetes, dyslipidemia, atrial fibrillation, carotid artery stenosis, obesity, hypercoagulability, and obstructive sleep apnea [16]. These conditions induce vascular endothelial injury, promote thrombosis, or cause arterial stenosis, ultimately leading to focal cerebral ischemia [17,18]. Upon vascular occlusion, the acute interruption of cerebral blood supply initiates an energy crisis and activates a cascade of oxidative stress-driven pathological processes [19,20].

**Fig. 1:**
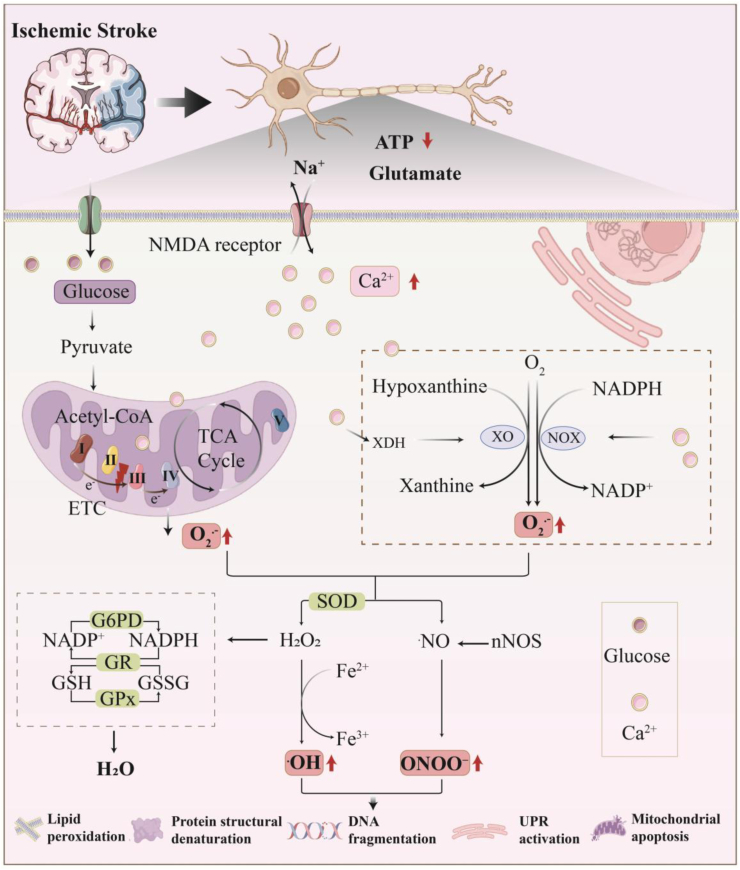
**ROS and RNS generation in neurons during ischemic stroke**. Ischemic stroke induces ATP depletion, triggering massive glutamate release that activates NMDA receptors and ultimately leads to calcium ion influx. Ca^2+^ Overload induces mitochondrial electron transport chain dysfunction and electron leakage, directly generating the O_2_•^-^. Concurrently, Ca^2+^ activates NADPH oxidase, which consumes oxygen to produce O_2_•^-^. Furthermore, Ca^2+^-activated proteases promote the conversion of XDH to XO. XO utilizes hypoxanthine and oxygen to react and generate O_2_•^-^. H_2_O_2_ and Fe^2+^ react via the Fenton reaction to produce the highly destructive •OH. In the reactive nitrogen pathway, the Ca^2+^-calmodulin complex activates neuronal nNOS to produce ˙NO. ˙NO rapidly combines with O_2_•^-^ to form the potent oxidant and nitrating agent, ONOO^−^. To counter this oxidative stress, cells activate a multi-tiered defense system: 1) SOD catalyzes the conversion of O_2_•^-^ to H_2_O_2_. 2) G6PD generates NADPH, which is then used by GR to regenerate reduced GSH from oxidized glutathione. This regenerated GSH is consumed by GPx to decompose H_2_O_2_ into H_2_O, collectively maintaining cellular redox homeostasis. When these defense systems are overwhelmed, accumulated ROS and RNS attack critical cellular structures: lipid peroxidation of cell membranes; denaturing and inactivating proteins; causing DNA fragmentation; promoting mitochondrial apoptosis and activating the endoplasmic reticulum UPR. Together, these processes drive neuronal damage and apoptosis.ROS, Reactive oxygen species; RNS, Reactive nitrogen species; O_2_•^-^, Superoxide anion; H_2_O_2_, Hydrogen peroxide; •OH, Hydroxyl radical; NO, Nitric oxide; ONOO^−^, Peroxynitrite anion; nNOS, Neuronal nitric oxide synthase; XDH, Xanthine dehydrogenase; XO, Xanthine oxidase; UPR, Unfolded protein response. SOD, Superoxide dismutase; G6PD, Glucose-6-phosphate dehydrogenase; GR, Glutathione reductase; GSH, Glutathione; GSSG, Glutathione disulfide; GPx, Glutathione peroxidase.

The essence of oxidative stress lies in the imbalance between oxidant generation and the antioxidant defense system [21]. Free radicals, characterized by their unpaired electrons and high reactivity, are primarily categorized into ROS and reactive nitrogen species (RNS) [22]. Fig. 1 summarizes the primary mechanisms of ROS and RNS production within neurons in ischemic stroke. Mitochondria are the primary intracellular source of ROS [23]. Under ischemic conditions, oxygen and nutrient deprivation inhibit the tricarboxylic acid cycle (TCA cycle) and oxidative phosphorylation, leading to a sharp decrease in ATP synthesis [24]. Simultaneously, dysfunction of the ETC causes electron leakage at complexes I and III, generating superoxide anion (O_2_•^-^). Furthermore, NADH accumulation, acidosis, and calcium overload amplify mitochondrial ROS production [25]. Upon reperfusion, the influx of oxygen reacts with the highly reduced components of the ETC intensely. This drives reverse electron transfer (RET) at complex I via succinate, resulting in a burst generation of O_2_•^-^ [26]. Beyond mitochondria, xanthine oxidase (XO) and NADPH oxidase (NOX) are also significant sources of ROS [27]. During ischemia, ATP depletion leads to hypoxanthine accumulation. Activated by calcium, xanthine dehydrogenase (XDH) converts into active XO. At reperfusion, XO catalyzes hypoxanthine to uric acid utilizing oxygen [26,28]. During this process, single-electron leakage directly generates O_2_•^-^, which can be further converted to hydrogen peroxide (H_2_O_2_) and highly oxidizing hydroxyl radical (•OH) [29]. Concurrently, NOX is activated by inflammatory factors and calcium. They transfer electrons from NADPH to oxygen via a transmembrane electron transport chain, producing O_2_•^-^ directly [29,30].

However, ATP depletion compromised the crucial antioxidant defense system. Lacking ATP, the core enzyme of the glutathione (GSH) system, glutathione reductase (GR) fails to reduce oxidized glutathione (GSSG) back to GSH. Simultaneously, the NADPH-dependent pentose phosphate pathway is impaired, further weakening GSH regeneration capacity [31]. This renders the GSH-dependent glutathione peroxidase (GPX) unable to clear H_2_O_2_ and lipid peroxides, triggering cellular oxidative damage directly [27]. Notably, although superoxide dismutase (SOD) continues to catalyze the conversion of O_2_•^-^ to H_2_O_2_ during ischemia/reperfusion, the downstream GSH system paralysis prevents the effective clearance of the generated H_2_O_2_. Instead, this accumulated H_2_O_2_ converts into the highly toxic •OH, exposing cells to a devastating oxidative storm during reperfusion [25]. Ultimately, the massive production of ROS directly oxidizes membrane lipids, modifies proteins, and damages DNA, leading to the loss of membrane integrity [25]. Critically, ROS also induces mitochondrial and cellular damage, releasing DAMPs, particularly mitochondrial components. These DAMPs exacerbate neuronal injury by activating pattern recognition receptors and triggering the release of inflammatory cytokines [32,33].

In ischemic stroke, oxidative stress acts as both an executor of damage and a signal amplifier. It dynamically interacts with energy metabolism disturbances, excitotoxicity, BBB disruption, neuroinflammation, and cell death pathways. Together, these processes accelerate brain tissue destruction and lead to severe neurological deficits.

## 3 Mitochondrial fragments release and functional mitochondria transfer

Mitochondria play a central role in the pathogenesis of ischemic stroke by mediating energy metabolism dysfunction and neuroinflammation [34,35]. Under physiological conditions, the OMM contains porins that permit the passage of small molecules and ions, while restricting larger molecules such as Cyt *c* and mtDNA [36]. The IMM, characterized by its high impermeability, regulates transmembrane transport through specific carrier proteins that enable selective molecular exchange. These tightly regulated mechanisms preserve mitochondrial integrity and maintain internal homeostasis. Ischemic stroke compromises mitochondrial membrane integrity, allowing mitochondrial components to escape through permeabilization pathways involving the mPTP, BAK/BAX oligomerization, and GSDMD-NT pore formation [37,38]. The release of dysfunctional mitochondrial fragments activates downstream PRRs, contributing to neuroinflammatory cascades [37,39].

The release of dysfunctional mitochondrial fragments by damaged cells serves as a sensitive indicator of cellular distress, transmitting signals to surrounding cells and initiating the transfer of functional mitochondria to injured cells. In cerebral ischemia/reperfusion (CI/RI) models, neurons release damaged mitochondria that are recognized by astrocytes as a “help me” signal [11]. This recognition leads to a marked increase in the expression of mitochondrial Rho GTPase 1 (Miro1) and TFAM mRNA in astrocytes, promoting the formation of TNTs between neurons and astrocytes [11].

Functional mitochondria transfer refers to the process by which mitochondria are transferred from one cell to another, serving to maintain cellular homeostasis and respond to pathological stress [12]. This phenomenon facilitates the redistribution of mitochondria across cellular boundaries, enabling cells with abundant and functional mitochondria to donate them to recipient cells experiencing mitochondrial dysfunction or increased energy demand. Through this mechanism, cells regulate energy metabolism, redox balance, and signal transduction pathways [40,41]. The transfer of functional mitochondria occurs via multiple intercellular routes, including TNTs, EVs, and direct cell fusion [12]. In ischemic stroke, astrocytes and MSCs have been shown to deliver functional mitochondria to injured neurons [42]. These transferred mitochondria rapidly restore the energy supply, enhance ATP synthesis, alleviate oxidative stress, and prevent apoptosis in energy-deprived neurons [13,43].

## 4 Dysfunctional mitochondrial fragments in the NVU

The NVU, consisting of neurons, astrocytes, pericytes, and vascular endothelial cells, plays a central role in regulating cerebral blood flow, maintaining BBB integrity, and providing metabolic support to neurons. Under pathological conditions such as cerebral ischemia, hypoxia, or oxidative stress, dysfunctional mitochondrial fragments are released into the extracellular space through various pathways, including mPTP opening, BAK/BAX oligomerization, and GSDMD-mediated pore formation. Mitochondrial components such as mtDNA, ROS, cytochrome *c*, and ATP act as DAMPs, activating PRRs including the cGAS-STING axis, NLRP3 and AIM2 inflammasomes, and TLRs, thereby initiating inflammatory cascades. Ultimately, the activation of these pathways promotes disease progression by amplifying neuroinflammation, disrupting BBB integrity, and inducing neuronal cell death.

### 4.1 Release of mitochondrial fragments

Mitochondria, the central hub of energy metabolism in eukaryotic cells, enclose numerous functional molecules within the matrix and cristae, bounded by a double membrane structure. These organelles regulate cellular energy homeostasis and fate through dynamic and tightly controlled signaling networks [44]. The oxidative phosphorylation (OXPHOS) system on the IMM generates a proton gradient via the ETC, which is utilized by ATP synthase (Complex V) to produce ATP. This process supplies approximately 90 % of the cellular energy under aerobic conditions [45]. During electron transport, Cyt *c, a* soluble electron carrier localized in the mitochondrial intermembrane space, shuttles electrons from Complex III to Complex I, ensuring efficient electron flow through the ETC [46]. mtDNA, a circular double-stranded genome, encodes 13 essential subunits of the respiratory chain (including key components of complexes I, III, IV, and ATP synthase), 22 tRNAs, and 2 rRNAs. Due to its lack of histone protection and limited DNA repair capacity, mtDNA is highly vulnerable to oxidative stress. Accumulation of mtDNA mutations is closely linked to neurodegenerative disorders [47,48]. Metal ion homeostasis is essential for mitochondrial function. Iron-sulfur clusters (ISCs) assembled by iron ions (Fe^2+^/Fe^3+^) are cofactors for ETC complexes, and copper ions (Cu^+^/Cu^2+^) are specifically incorporated into the catalytic center of COX [49,50], regulated by ΔΨm and transporters such as mitoferrin. Disturbances in ion homeostasis induce O_2_•^-^ bursts and OXPHOS dysfunction [51,52].

Under physiological conditions, transient mPTP, formed by the ANT, VDAC, and cyclophilin D (Cyp D), opening participates in Ca^2+^ buffering and redox homeostasis. [52]. However, pathological stimuli such as Ca^2+^ overload, mtROS burst, or ATP depletion leads to sustained and irreversible mPTP opening, resulting in mitochondrial matrix swelling, outer membrane rupture, and release of mitochondrial contents such as ATP, mtROS, mtDNA, Cyt *c*, and free metal ions (e.g., Fe^3+^), activating cytoplasmic PRRs and initiate cell death pathways [[53], [54], [55]]. In response to apoptotic signals such as DNA damage or growth factor withdrawal, the pro-apoptotic Bcl-2 family proteins BAK and BAX undergo conformational activation, oligomerize, and insert into the OMM, forming nanoscale pores [56]. These pores permit the efflux of molecules ≤5 kDa (e.g., Cyt *c*) into the cytosol driven by concentration gradients, activating the caspase cascade and initiating programmed cell death [57]. BAK/BAX pores exhibit dynamic plasticity, with pore assembly, size, and dynamics modulated by BH3-only proteins [58]. During pyroptosis, caspases 1, 4, 5, and 11 cleave GSDMD, releasing its N-terminal domain (GSDMD-NT). GSDMD-NT oligomerizes to form nanometer-scale β-barrel-shaped pores (typically ∼10–20 nm in diameter) that primarily insert into the plasma membrane. This disrupts ion homeostasis, leading to cell swelling and allowing the passive release of inflammatory cytokines such as IL-1β [59]. Emerging evidence also suggests that gasdermin E (GSDME), upon cleavage by caspase-3, binds to mitochondrial cardiolipin and forms pores in the mitochondrial inner membrane [60]. This disrupts mitochondrial integrity, leading to mitochondrial outer membrane permeabilization (MOMP), release of mtDNA, and ultimately amplifying inflammatory signaling, particularly if the cell lyses. This capacity of different gasdermin family members (GSDMD, GSDME) to form pores in distinct cellular membranes (plasma membrane, mitochondrial membranes) highlights their functional versatility in regulating cell death and inflammation.

Dysfunctional mitochondrial fragments are expelled into the extracellular space via EVs actively [61]. For instance, mitophagy, the selective degradation of damaged mitochondria through the PTEN-induced putative kinase 1 (PINK1) and Parkin RBR E3 ubiquitin-protein ligase (Parkin) pathway, serves as a key quality control mechanism. Impairment of this pathway results in the pathological accumulation of damaged mitochondria, increasing the risk of membrane rupture and cytoplasmic leakage [62]. In addition, defects in lysosomal acidification or protease activity hinder autophagosome maturation and degradation, leading to the accumulation of mitochondria-containing autophagosomes. These autophagosomes may subsequently fuse with the plasma membrane, enabling the extracellular release of mitochondrial components [63,64]. In contrast, passive mitochondrial release primarily occurs during cell injury or death processes, including apoptosis, necrosis, and pyroptosis [65].

### 4.2 Mitochondrial·fragments activate PRRs

**Fig. 2:**
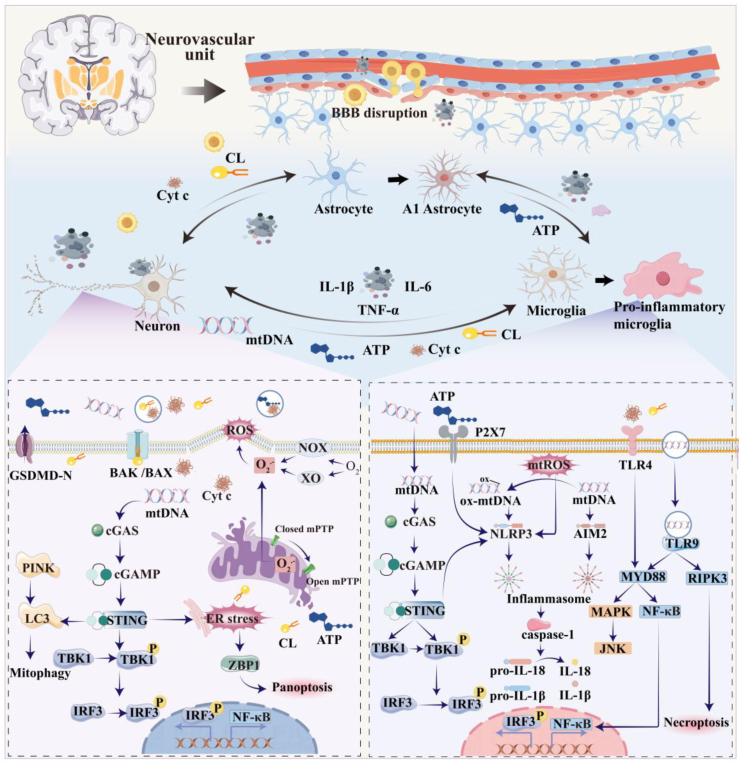
**Dysfunctional mitochondrial fragments in the NVU.** Stimulation derived from plasma membrane NOX, cytoplasmic XO, and mitochondrial ROS triggers the opening of the mPTP, leading to the release of mitochondrial contents, mitochondrial fragmentation, and even cell death. Neuronal mtDNA activates the cGAS-STING pathway within neurons, inducing ER stress and triggering panoptosis, while simultaneously promoting mitophagy. Neuron-derived dysfunctional mitochondrial fragments, including mtDNA, ox-mtDNA, ATP, and CL, synergize with astrocyte-derived ATP to activate microglial PRRs: cGAS-STING axis, NLRP3/AIM2 inflammasomes, and TLRs. Activation of these PRRs induces microglia polarization toward the pro-inflammatory microglial phenotype, characterized by upregulation of IL-1β and TNF-α. Affected microglia generate mtROS and oxidize mtDNA to form ox-mtDNA. Both mtROS and ox-mtDNA directly activate the NLRP3 inflammasome. Furthermore, mitochondrial fragments released by microglia promote the transformation of astrocytes into the neurotoxic A1 phenotype. A1 astrocytes and M1 microglia cooperatively exacerbate neuroinflammation by releasing pro-inflammatory mediators such as IL-6, thereby intensifying neuronal apoptosis and BBB disruption.NVU, Neurovascular unit; mtDNA, Mitochondrial DNA; cGAS-STING, Cyclic GMP-AMP synthase–stimulator of interferon genes; ER, Endoplasmic reticulum; ox-mtDNA, Oxidized mitochondrial DNA; ATP, Adenosine triphosphate; CL, Cardiolipin; PRRs, Pattern recognition receptors; NLRP3, NLR family pyrin domain containing 3; AIM2, Absent in melanoma 2; TLRs, Toll-like receptors; IL-1β, Interleukin-1 beta; TNF-α, Tumor necrosis factor-alpha; Cyt *c*, Cytochrome *c*; IL-6, Interleukin-6; BBB, Blood-brain barrier; NOX, NADPH oxidase; XO, xanthine oxidase.

Fig. 2 depicts dysfunctional mitochondrial fragments triggers neuroinflammatory responses by activating multiple PRRs, including the cGAS-STING axis, NLRP3/AIM2 inflammasomes, and TLRs. These pronounced activate microglia and astrocytes, while concurrently disrupting the integrity of the BBB. The resulting inflammatory cascade promotes sustained production of pro-inflammatory mediators and neurotoxic factors, ultimately contributing to neuronal degeneration and cell death.

#### 4.2.1 cGAS-STING

The cGAS-STING pathway functions as a pivotal sensor of cytosolic DNA and represents a key link between mitochondrial dysfunction and neuroinflammation. cGAS possesses a nucleotidyltransferase catalytic domain and two DNA-binding domains, maintaining the enzyme in an autoinhibited conformation under resting conditions [66,67]. Upon binding to mtDNA released into the cytoplasm, cGAS undergoes dimerization and conformational rearrangement, enabling it to catalyze the conversion of ATP and guanosine triphosphate (GTP) into 2′3′-cyclic GMP-AMP (cGAMP), a unique cyclic dinucleotide containing both 2′–5′ and 3′–5′ phosphodiester linkages [68,69]. cGAMP is a second messenger that binds to the adaptor protein STING located on the endoplasmic reticulum (ER) [69,70]. Upon activation, STING translocates from the ER through the ER-Golgi intermediate compartment to the Golgi apparatus (Golgi) [71,72]. During this trafficking process, STING recruits and activates TANK-binding kinase 1 (TBK1) and IκB kinase (IKK), which phosphorylate and activate the transcription factors interferon regulatory factor 3 (IRF3) and nuclear factor kappa-light-chain-enhancer of activated B cells (NF-κB). These transcription factors then drive the expression of type I interferons and various pro-inflammatory cytokines (e.g., TNF-α, interleukin-6 (IL-6), IL-1β) [73,74].

As immune cells, microglia respond to abnormal DNA signals first in the central nervous system (CNS) [75]. Under various stress conditions, mitochondrial dysfunction in neurons leads to a loss of ΔΨm and subsequent leakage of mtDNA. Neuronal exosomes containing mtDNA are internalized by microglia, triggering activation of the cGAS-STING signaling pathway. This activation drives microglial polarization toward the pro-inflammatory M1 phenotype, characterized by the secretion of cytokines such as IL-1β, IL-6, and TNF-α [75,76]. Activated microglia transferred to astrocytes by releasing fragmented mitochondria. Upon uptake, these dysfunctional mitochondrial fragments induce astrocytic activation toward the A1 phenotype [77]. A1 astrocytes produce additional pro-inflammatory cytokines, lose neuroprotective functions, and release neurotoxic factors, exacerbating neuronal damage [77].

Neuronal mtDNA accumulates in the cytoplasm under stress conditions, activating the intracellular cGAS-STING signaling pathway. Following activation, the cGAS-STING complex is trafficked to lysosomes through interaction with microtubule-associated protein 1A/1B light chain 3 (LC3), facilitating a lysosome-dependent negative feedback loop that limits sustained type I IFN signaling [72,78,79]. STING-mediated autophagy contributes to the clearance of cytoplasmic DAMPs, including escaped mtDNA [80]. However, widespread activation of the cGAS pathway leads to excessive inflammation in pathological conditions, while the associated negative feedback mechanism is compromised due to lysosomal dysfunction. As a result, mtDNA accumulates in the cytoplasm, further amplifying the inflammatory response [80]. Recent studies have identified a regulatory interaction between STING and PTEN-induced PINK1, linking STING signaling to the control of mitophagy [81]. Notably, activation of the cGAS pathway is not solely detrimental; following peripheral nerve injury, cGAS activation has been associated with enhanced axonal regeneration, suggesting context-dependent neuroprotective or reparative effects [82].

#### 4.2.2 NLRP3 inflammasome

The NLRP3 inflammasome is one of the most extensively studied members of the inflammasome family and serves as a key cytoplasmic innate immune sensor. It responds to a series of DAMPs stimuli, initiating inflammasome assembly and triggering pro-inflammatory signaling cascades [83]. NLRP3 has been identified as a central mediator of neuroinflammation in numerous neurological disorders [84]. Studies have shown that the leakage of mtDNA into the cytoplasm of microglia activates the cGAS-STING pathway, which subsequently promotes NLRP3 inflammasome activation [85]. In addition, mtDNA in microglia is highly susceptible to oxidation by mtROS, forming oxidized mitochondrial DNA (ox-mtDNA). Subsequently, ox-mtDNA and microglial mtROS activates the NLRP3 inflammasome, which in turn induces the cleavage of caspase-1 and promotes the secretion of mature IL-1β [[86], [87], [88]]. Comparative analyses have demonstrated that ox-mtDNA exhibits stronger binding affinity to NLRP3 than unmodified mtDNA, facilitating direct interaction with the pyrin domain of NLRP3, a critical step for inflammasome activation [89]. ATP is the primary energy currency within cells, supporting a wide range of cellular processes. However, in certain neurodegenerative diseases, increased membrane permeability leads to the extracellular release of ATP from neurons and astrocytes. This extracellular ATP activates the P2X purinoceptor 7 (P2X7) receptor on microglia, promoting NLRP3 inflammasome activation and inducing the release of IL-1β and IL-18 [90,91].

#### 4.2.3 AIM2 inflammasome

As a key intracellular innate immune sensor, the AIM2 inflammasome is essential for recognizing cytosolic DNA derived from pathogens or damaged host cells. Detection of double-stranded DNA (dsDNA), including mtDNA, in the cytoplasm leads to inflammasome assembly, caspase-1 activation, and the proteolytic maturation of pro-inflammatory cytokines IL-1β and IL-18, amplifying the inflammatory response [92]. Notably, the release of unoxidized mtDNA in microglia activates the AIM2 inflammasome rather than the NLRP3 inflammasome [93]. This differential activation suggests distinct sensitivities of AIM2 and NLRP3 to the oxidation state of mtDNA, which may explain varying outcomes in studies of mtDNA leakage. Additionally, BAK activation induces the depletion of mitochondrial TFAM and mtDNA release, triggering AIM2 inflammasome. [94,95]. In the absence of cGAS, the AIM2 inflammasome serves as a compensatory mechanism to maintain immune surveillance in response to cytosolic mtDNA [96].

#### 4.2.4 TLRs

TLRs are pattern recognition receptors located on the cell surface or within endosomal compartments [97]. Signal transduction through TLRs occurs via two primary pathways: MyD88-dependent and MyD88-independent mechanisms. All TLRs except TLR3 utilize the MyD88 adaptor protein to initiate downstream signaling [98]. In addition to activating the cGAS-STING pathway following endocytic uptake into microglia, neuronal mtDNA also engages TLR9 within endosomes. TLR9 activation triggers NF-κB signaling, leading to the production of IL-1β, IL-18, and TNF-α [99]. Furthermore, TLR4 receptors expressed on microglia and astrocytes can be activated by exogenous mitochondrial components such as Cyt *c* and CL. Upon activation, TLR4 initiates the MyD88-dependent signaling cascade. This involves TLR4-MyD88 binding, recruitment of interleukin-1 receptor-associated kinases (IRAKs) and tumor necrosis factor receptor-associated factor 6 (TRAF6), followed by activation of the NF-κB and mitogen-activated protein kinase (MAPK) pathways. These events collectively enhance the transcription of inflammation-related genes and perpetuate neuroinflammatory responses [100,101].

## 5 Functional mitochondria transfer in ischemic stroke

Ischemic stroke is characterized by energy failure resulting from the interruption of cerebral blood flow [102]. Neurons, which possess the highest mitochondrial density among brain cell types, depend heavily on ATP generated through mitochondrial oxidative phosphorylation to support their metabolic demands [13,103]. Therefore, preserving mitochondrial function and restoring metabolic homeostasis represent critical therapeutic strategies. Fortunately, functional mitochondria can be transferred from healthy cells to injured neurons via intercellular structures such as TNTs and EVs. Transferred mitochondria support cellular recovery by attenuating oxidative stress, maintaining mitochondrial quality control, inhibiting apoptosis, restoring ATP production, and preserving BBB integrity.

### 5.1 Mechanisms of functional mitochondrial transfer

**Fig. 3:**
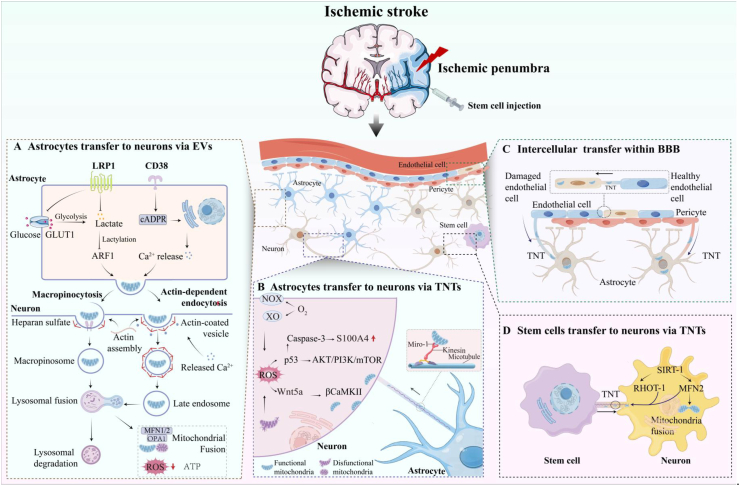
Mitochondria transfer in ischemic stroke. **(A) Astrocytes transfer to neurons via EVs:** Lactylation of ARF1 through CD38/cADPR signaling and LRP1 inhibition promotes functional mitochondria transfer via EVs. EVs internalization occurs through pinocytosis and endocytosis, followed by lysosomal degradation or fusion with damaged neuronal mitochondria. **(B) Astrocytes transfer to neurons via TNTs:** After ischemic stroke, ROS derived from plasma membrane NOX, cytoplasmic XO, and mitochondria initiate the formation of TNTs at the plasma membrane through activation of the Akt/PI3K/mTOR signaling pathway. ROS upregulates caspase-3 to guide the directional extension of TNTs by creating a spatial gradient of reduced S100A4. During the early stages of TNTs' elongation, Wnt5a-induced phosphorylation of βCaMKII causes its dissociation from actin filaments, enabling recruitment of actin-regulatory proteins and cytoskeletal assembly. The magnified panel highlights the structural components supporting mitochondrial trafficking within TNTs. **(C) Intercellular transfer within BBB:** Functional mitochondria are transferred from endothelial cells and pericytes to injured astrocytes via TNTs; Bidirectional mitochondrial exchange occurs between healthy and damaged endothelial cells through TNTs-mediated transfer. **(D) Stem cells transfer to neurons via TNTs:** Mesenchymal stem cells establish TNTs through SIRT-1-dependent upregulation of RHOT-1 and MFN2 expression. TNTs, Tunneling nanotubes; Akt, Protein kinase B; PI3K, Phosphoinositide 3-kinase; mTOR, Mechanistic target of rapamycin; S100A4, S100 calcium-binding protein A4; Wnt5a, Wingless-type MMTV integration site family member 5A; βCaMKII, β calcium/calmodulin-dependent protein kinase II; ARF1, ADP-ribosylation factor 1CD38, Cluster of differentiation 38; cADPR, Cyclic ADP-ribose; LRP1, Low-density lipoprotein receptor-related protein 1; EVs, Extracellular vesicles; SIRT-1, Sirtuin 1; RHOT-1 (or MIRO1), Ras homolog family member T1; MFN2, Mitofusin 2.

To date, the molecular and signaling mechanisms underlying mitochondrial transfer in ischemic stroke have not been fully elucidated. Most studies suggest that tunneling TNTs and EVs serve as the primary pathways for functional mitochondria transfer. However, significant gaps remain in our understanding of the key drivers that trigger functional mitochondria transfer between donor and recipient cells, as well as the subsequent cellular activities. By systematically reviewing existing evidence and synthesizing various molecular clues, this article aims to provide direction for future research into the underlying mechanisms of intercellular mitochondrial transfer. Fig. 3 summarizes the mechanism of functional mitochondrial transfer in ischemic stroke.

#### 5.1.1 Tunneling nanotubes

TNTs are dynamic, membrane-bound tubular structures that enable direct intercellular communication and cargo transport. Originating from the plasma membrane, TNTs form within minutes and maintain stable connections between cells for several minutes to hours [104,105]. TNTs are composed of cellular membrane structures supported by F-actin, myosin, and tubulin, which maintain their structural integrity and transport capacity. With diameters ranging from 50 to 1500 nm, TNTs transfer diverse cargos, including proteins, RNA, and organelles such as mitochondria and endoplasmic reticulum, to neighboring or distant cells across several hundred micrometers [106,107]. First identified in PC12 cells cultured *in vitro* in 2004, TNTs have been extensively investigated in multiple organs, including the heart, lungs, and kidneys [108]. In ischemic stroke, the mechanism by which TNTs transfer functional mitochondria has been extensively studied.

Ischemia impairs mitochondrial energy metabolism and disrupts the ETC, leading to electron leakage and excessive generation of ROS, such as O_2_•^-^, H_2_O_2,_ and •OH. H_2_O_2_ activates p38 MAPK in neurons, triggering a cascade that enhances the activity of the tumor suppressor protein p53, a central regulator of TNTs biogenesis [[109], [110], [111]]. p53 promotes TNTs formation through two mechanisms. First, it upregulates the epidermal growth factor receptor (EGFR), which activates the phosphoinositide 3-kinase (PI3K)/Akt/mammalian target of the rapamycin (mTOR) pathway [112]. This signaling cascade induces overexpression of M-Sec (TNFAIP2), which cooperates with RAS-like protein A (RalA) and the exocyst complex, facilitates actin polymerization and TNTs assembly [13,[113], [114], [115]]. Notably, TNF-α independently enhances M-Sec expression via NF-κB activation [109,116]. Second, p53-mediated activation of caspase-3 promotes the proteolytic cleavage of S100 calcium-binding protein A4 (S100A4). The resulting S100A4 gradient between stressed donor cells and recipient cells provides directional cues for TNTs extension [109,117,118]. Moreover, CI/RI upregulates neuronal wingless-type mmtv integration site family, member 5a (Wnt5a), inducing transient calcium/calmodulin-dependent protein kinase II beta (βCaMKII) phosphorylation. This phosphorylation event causes βCaMKII to dissociate from actin filaments, allowing actin-regulatory proteins to be recruited for rapid TNTs assembly. Subsequent dephosphorylation of βCaMKII enables its reattachment to actin, stabilizing the cytoskeleton and prolonging TNTs lifespan [119]. This dynamic phosphorylation–dephosphorylation cycle ensures both the efficient formation and structural maintenance of TNTs [120]. Additionally, myosin X (Myo10) in neurons also contributes to TNTs formation via a pathway independent of Akt activation. Unlike βCaMKII, Myo10 enhances cargo delivery from donor to recipient cells, likely due to its intrinsic cargo-binding properties [121]. Functional mitochondria transfer through TNTs depends on the actin cytoskeleton and intracellular trafficking proteins. Miro1 and Miro2, Rho GTPases located on the outer mitochondrial membrane, form complexes with motor proteins through trafficking kinesin proteins 1 and 2 (TRAK1/2), facilitating mitochondrial movement along microtubules, an essential process also observed in axonal transport. In astrocytes and neurons, high levels of Miro1/2 in donor cells significantly enhance functional mitochondria transfer efficiency, while alterations in Miro1/2 levels in recipient cells appear to have minimal impact on the process [122,123].

Experimental evidence demonstrates that coculture of MSCs with hypoxic neuronal cells induces a time-dependent upregulation of SIRT-1 expression [124]. A key interaction between mitofusin 2 (MFN2) and RHOT 1 facilitates the attachment of mitochondria to motor proteins, thereby enhancing mitochondrial motility [125]. Consistent with these findings, the formation of TNTs is markedly reduced in the presence of SIRT-1 inhibitors, underscoring the essential roles of MFN2 and RHOT1 in TNT-mediated functional mitochondria transfer. Elevated SIRT-1 expression also upregulates peroxisome proliferator-activated receptor gamma coactivator 1-alpha (PGC-1α) [124], promoting mitochondrial biogenesis. Simultaneously, the SIRT-1/MFN2 signaling axis contributes to the regulation of mitochondrial homeostasis, playing a central role in mitochondrial quality control [[126], [127], [128], [129]]. Astrocytes are capable of acquiring functional mitochondria from pericytes and endothelial cells via TNTs [130]. Similarly, TNT-mediated functional mitochondria transfer has been observed between healthy endothelial cells and those with mitochondrial dysfunction [131]. However, the specific mechanisms governing TNTs formation remain incompletely characterized.

#### 5.1.2 Extracellular vesicles

EVs are heterogeneous membrane particles secreted by all cell types, classified into three categories by biogenesis, size, and composition: microvesicles (100 nm-1 μm), exosomes (30–100 nm), and apoptotic bodies (1–2 μm) [132,133]. Studies reveal that EVs deliver functional mitochondria to recipient cells, restoring bioenergetics in damaged cell [134,135]. The mechanisms underlying EVs formation and internalization are summarized.

Ischemia/hypoxia-induced energy deficit disrupts ion homeostasis, culminating in Ca^2+^ overload via activation of L-type voltage-gated Ca^2+^ channels [136,137]. This pathological Ca^2+^ surge induces glutamate release from neurons, which stimulates astrocytic CD38 upregulation. CD38 catalyzes the synthesis of cyclic ADP-ribose (cADPR), enhancing Ca^2+^ release through ryanodine receptor (RyR) activation [[138], [139], [140]]. Through this CD38/cADPR signaling cascade, astrocytes initiate EVs-mediated transfer of functional mitochondria to neurons, thereby restoring neuronal bioenergetics and improving cell survival [15]. Further studies suggest that the maintenance of mitochondrial function during EVs-mediated transfer may involve CD38-induced enhancement of mitochondrial protein O-Linked Β-N-Acetylglucosaminylation (O-GlcNAcylation), a post-translational modification regulated by ER and Golgi trafficking. Inhibition of this trafficking by Brefeldin A suppresses O-GlcNAcylation, indicating its dependence on intracellular vesicular transport [141]. Notably, this O-GlcNAcylation process is impaired under pathological conditions such as chronic inflammation and aging, primarily due to the downregulation of solute carrier family 35 member B4 (SLC35B4), a critical regulator of glycosylation pathways [141]. Recent studies have identified another pathway for astrocyte-to-neuron functional mitochondria transfer. Low-density lipoprotein receptor-related protein 1 (LRP1) is a key regulator of metabolic reprogramming in astrocytes following ischemia-reperfusion injury. Downregulation of LRP1 shifts astrocytic metabolism toward glycolysis, leading to lactate accumulation and subsequent protein lactylation, particularly of ADP-ribosylation factor 1 (ARF1). Analysis of ARF1 interacting proteins reveals enrichment in vesicle budding and trafficking pathways, suggesting that lactylated ARF1 coordinates EVs mediated mitochondrial export [142].

The mechanisms by which EVs containing functional mitochondria are internalized and integrated into recipient cells following ischemic stroke remain incompletely understood. Current evidence suggests that EVs uptake primarily occurs through endocytic pathways [143,144]. However, the precise mode of endocytosis remains under debate. Studies using specific inhibitors targeting different endocytic routes have provided insights into the internalization mechanisms. For example, cardiomyocytes treated with inhibitors of clathrin-mediated endocytosis (methyl-β-cyclodextrin), actin-mediated endocytosis (cytochalasin D), macropinocytosis [5-(N-ethyl-N-isopropyl)-amiloride], and microtubule polymerization (nocodazole) demonstrated that mitochondrial uptake is primarily dependent on actin-driven endocytosis [145]. Similarly, treatment of HepG2 and fibroblast cells with macropinocytosis inhibitors (amiloride or 5-(N-ethyl-N-isopropyl)-amiloride) and clathrin-mediated endocytosis blockers (hypertonic sucrose, potassium depletion, or cytosolic acidification) revealed that inhibition of macropinocytosis suppressed mitochondrial uptake in a dose-dependent manner, whereas clathrin inhibition had no significant effect [146].

Macropinocytosis, a form of pinocytosis, is an actin-dependent endocytic process characterized by the formation of large intracellular vacuoles. Recognized as a widespread and evolutionarily conserved mechanism, macropinocytosis has been proposed to have played a role in the early mitochondrial endosymbiosis event [147,148]. Its relevance in mitochondrial uptake is supported by studies demonstrating that MSCs and hepatocytes internalize isolated mitochondria predominantly through this pathway [[149], [150], [151]]. Macropinocytosis is regulated by extracellular environmental signals and does not rely on specific receptor-ligand interactions, allowing for non-selective uptake across various cell types [148,152]. Interestingly, HepG2 and fibroblast cells exhibit selective uptake of mitochondria possessing intact outer membranes, with the internalization process requiring interaction with cellular heparan sulfate proteoglycans [146]. After internalization, extracellular mitochondria are trafficked through the endosomal-lysosomal system. Mostly, exogenous mitochondria escape from endosomes and lysosomes successfully, and subsequently, they fuse with the endogenous mitochondrial network, as observed in cardiomyocytes [144]. The fusion process is facilitated by MFN1/2 and optic atrophy 1 (OPA1), which play key roles in mitochondrial membrane dynamics in both cardiomyocytes and fibroblasts [153]. However, the precise mechanisms by which exogenous mitochondria escape from endosomal and lysosomal compartments remain unclear.

#### 5.1.3 Trigger signals

The factors influencing functional mitochondria transfer encompass four aspects: donor cells, recipient cells, transfer vehicles, and the *in vivo* microenvironment. In ischemic stroke, disruption of redox balance leads to massive generation of ROS such as O_2_•^-^, H_2_O_2_, •OH, and ONOO^−^, which exert complex effects on functional mitochondria transfer. Most studies indicate that high energy-demanding cells serving as mitochondrial recipients are prone to accumulating stress-induced ROS, triggering intercellular rescue transfer of functional mitochondria [43,154]. Compared to untreated cells, recipients subjected to H_2_O_2_ interference contain higher mitochondrial content derived from the donor. Notably, eliminating intracellular ROS in recipient cells using the scavenger N-acetylcysteine (NAC) significantly reduces functional mitochondria transfer [155]. Concurrently, when recipient cells produce excessive ROS, their internal mitochondria become damaged. As distress signals transmitted to neighboring astrocytes, these dysfunctional mitochondria promote directional transfer of functional mitochondria from astrocytes to damaged neurons [11]. This phenomenon is associated with H_2_O_2_-mediated facilitation of TNTs formation between recipient and donor cells. Studies demonstrate that H_2_O_2_ significantly enhances actin polymerization and the binding of myosin Va to F-actin filaments between astrocytes and neurons [110]. Crucially, compared to ROS-free recipient cells, ROS-producing cells exhibit not only higher TNTs formation efficiency but also increased internalization of exogenous mitochondria within 24 h post-OGD/R, correlating with rising ROS levels [11,43,156,157].

However, it is emphasized that current evidence supporting these mechanisms primarily originates from *in vitro* models. While such models elucidate fundamental principles, they fail to fully replicate the complex *in vivo* microenvironment following ischemic stroke—particularly sustained inflammatory responses and oxidative stress. This pathological milieu may profoundly impact key processes of functional mitochondria transfer, including: Ⅰ) Transfer capacity of donor cells; Ⅱ) Formation and stability of transfer vehicles (e.g., TNTs or EVs); Ⅲ) Uptake and functional integration of transferred mitochondria by recipient cells. Although some aforementioned studies suggest elevated ROS levels promote functional mitochondria transfer, it remains unclear whether excessive inflammatory signals or ROS adversely affect the structural integrity of TNTs and EVs. This concern arises from the critical role of lipids in maintaining structural stability of EVs and TNTs, coupled with the established fact that ROS oxidize lipids [158]. Therefore, deepening our understanding of how inflammation and oxidative stress regulate the functional mitochondria transfer machinery is essential for evaluating its *in vivo* therapeutic efficacy and translational potential.

### 5.2 Functions of mitochondria transfer

In ischemic stroke, the transfer of functional mitochondria to damaged cells inhibits oxidative stress, maintains mitochondrial quality homeostasis, restores neuronal energy metabolism, suppresses apoptosis, and preserves BBB integrity.

#### 5.2.1 Inhibition of oxidative stress

In ischemic stroke, oxidative stress is both an executor of damage and an amplifier of signaling cascades. As the primary site of ROS generation, mitochondria critically determine the extent of cellular injury through functional status. Functional mitochondria transfer exerts multidimensional antioxidant effects, with core mechanisms encompass: restoration of energy metabolic homeostasis, direct scavenging of ROS, and mitophagy.

Following the acquisition of functional mitochondria via TNTs or EVs, recipient cells demonstrate enhanced ATP biosynthesis capacity through functional mitochondrial integration. This process optimizes ETC functionality by preserving the integrity of Complexes I-IV, reducing O_2_•^-^ accumulation caused by electron leakage and mitigating ROS overproduction following energy failure [124,159]. For instance, in ischemic stroke models, stem cell-derived functional mitochondria transfer to neurons significantly elevates intracellular ATP levels, and enhances activities of respiratory enzymes including citrate synthase (CS), pyruvate dehydrogenase (PDH), and α-ketoglutarate dehydrogenase (α-KGDH), while concurrently reducing ROS concentrations [124]. Moreover, transferred mitochondria deliver endogenous antioxidant enzymes such as SOD and glutathione peroxidase (GSH-Px), while synergistically providing reducing equivalents to maintain redox equilibrium. Functional mitochondria transfer has been shown to upregulate GSSG levels and decrease the GSH/GSSG ratio, activating glutathione metabolic pathways to suppress intracellular ROS accumulation [160]. Additionally, emerging evidence suggests two potential mechanisms: transferred functional mitochondria activate the PINK1/Parkin pathway to promote mitophagy, eliminating fragmented mitochondria and interrupting persistent ROS release [161]; or fuse with damaged mitochondria to reduce the population of ROS-generating organelles. Current research on the antioxidant mechanisms of functional mitochondria transfer in ischemic stroke still exhibits significant knowledge gaps, with existing evidence predominantly focused on phenomenological descriptions and lacking in-depth analysis of specific regulatory mechanisms.

Although functional mitochondria transfer is recognized to mitigate ROS, most evidence remains confined to *in vitro* studies. In complex pathological settings *in vivo*, further research is needed to elucidate: (Ⅰ) the extent to which such transfer reduces endogenous ROS levels, and (Ⅱ) whether functional mitochondria transfer occurs under conditions of elevated ROS.

#### 5.2.2 Maintaining mitochondrial quality control

MQC is essential for preserving mitochondrial function and cellular homeostasis [162]. It encompasses a network of regulatory processes, including mitochondrial biogenesis, fission, and fusion dynamics, and mitophagy [62]. Together, these mechanisms ensure adequate energy production, structural integrity, and the timely removal of damaged mitochondria.

As a critical compensatory response in ischemic stroke, mitochondrial biogenesis reduces cerebral blood flow, leading to an insufficient neuronal energy supply. In this context, neurons adapt by increasing mitochondrial content to meet elevated energy demands [163]. When intrinsic mitochondrial biogenesis is inadequate, the recruitment of mitochondria from neighboring cells may serve as an alternative compensatory mechanism. The formation of TNTs, typically initiated by metabolically stressed or damaged cells, enables the directed transport of functional mitochondria from donor to recipient cells, a process that may supplement or partially replace endogenous mitochondrial biogenesis [163].

In ischemic stroke, astrocytes have been shown to transfer functional mitochondria to neurons via CD38-mediated EVs, restoring neuronal viability. Furthermore, stem cells transfer functional mitochondria to neurons through TNTs, resulting in increased neuronal ATP production [15,124,164]. A common feature in these processes is the elevated mitochondrial content observed in recipient neurons following intercellular functional mitochondria transfer. Beyond neuron-targeted interactions, functional mitochondrial exchange between pericytes and astrocytes restores astrocytic function. Healthy pericytes are also capable of transferring functional mitochondria to relatively compromised pericytes, contributing to the maintenance of vascular and metabolic homeostasis [130].

Transferred mitochondria represent a small portion of the recipient cell's overall mitochondrial network. Activation of mitochondrial biogenesis may be more critical in sustaining energy metabolism. Healthy exogenous mitochondria can promptly initiate oxidative phosphorylation to mitigate acute energy deficits and subsequently proliferate within recipient cells, triggering mitochondrial biogenesis and supporting long-term energy restoration. Recent studies have shown that coculture of stem cells with hypoxic PC12 cells increases SIRT-1 expression in PC12 cells, which upregulates PGC-1α and promotes mitochondrial biogenesis. These findings provide further support for the role of functional mitochondria transfer in activating endogenous biogenic pathways [130]. However, in the post-ischemic inflammatory environment, signaling pathways activating key biosynthetic regulators such as PGC-1α may be suppressed. The activation of inflammatory factors like NF-κB is typically associated with downregulation of PGC-1α [165]. Thus, whether transferred functional mitochondria effectively initiate endogenous biosynthesis programs under intense inflammatory conditions requires further validation through *in vivo* studies.

Mitochondria are morphologically dynamic organelles that undergo continuous fission and fusion, essential for regulating mitochondrial integrity, bioenergetic function, and cellular homeostasis under physiological and pathological conditions [166]. Under normal circumstances, mitochondrial shape, size, and number adapt through balanced fusion and fission to meet cellular metabolic demands. However, in response to ischemic and hypoxic injury, mitochondrial oxidative stress disrupts the mitochondrial fission/fusion balance, promoting excessive fragmentation and increasing neuronal vulnerability to cell death.

Dynamin-related protein 1 (DRP1), a key regulator of fission, is recruited to the outer mitochondrial membrane by several receptor proteins, including mitochondrial fission protein 1 (Fis1), 49 and 51-kDa mitochondrial dynamin-like proteins, and the mitochondrial fission factor. DRP1 oligomerizes and interacts with dynamin 2 to form a constrictive ring-like structure, ultimately severs the mitochondrial membrane through GTP hydrolysis and mechanical constriction [[167], [168], [169]]. In ischemic stroke, elevated DRP1 expression enhances mitochondrial fragmentation, an event widely recognized as an early driver of neuronal cell death [170,171]. However, Sarmah et al. revealed that DRP1 expression was reduced in hypoxic PC12 cells cocultured with stem cells, compared to hypoxic PC12 cells cultured alone. These observations suggest that functional mitochondria transfer may downregulate DRP1 expression, inhibiting excessive fission and preserving mitochondrial integrity under hypoxic stress [124].

Mitochondrial fusion facilitates the exchange of matrix contents and metabolites, such as proteins, mtDNA, or membrane components, essential for the ETC [172]. Fusion also enables damaged mitochondria to restore function by merging with healthy mitochondria, allowing functional complementation and repair [173]. Key fusion-related proteins, including OPA1 and MFN1/2, play protective roles in ischemic neurons by promoting mitochondrial network integrity [174]. Disruption of mitochondrial fusion, either through OPA1 downregulation or MFN2 depletion, has been shown to impair intracellular homeostasis and exacerbate neuronal injury during ischemic stroke. In contrast, functional mitochondria transfer has been found to increase the expression of MFN1 and MFN2 in hypoxic PC12 cells, thereby promoting mitochondrial fusion and supporting cellular recovery [124]. Notably, the healthy mitochondria that participate in fusion with damaged mitochondria are not necessarily endogenous; instead, they may originate from donor cells, highlighting the significance of exogenous functional mitochondria transfer in maintaining mitochondrial dynamics and neuronal viability [15].

#### 5.2.3 Neuronal energy metabolism restoration

Cellular energy metabolism encompasses the biochemical processes by which cells generate and utilize energy to sustain essential physiological functions. Mitochondria, often referred to as the powerhouses of the cell, serve as the primary sites of ATP production. In the nervous system, ATP is primarily generated through glycolysis and oxidative phosphorylation pathways [175]. Glycolysis converts glucose into pyruvate in the cytoplasm, yielding a modest amount of ATP. Pyruvate then enters the mitochondria, where it is further oxidized through the TCA cycle and the oxidative phosphorylation pathway, producing the majority of cellular ATP [176]. Among these two processes, oxidative phosphorylation remains the predominant source of energy in neurons due to their high metabolic demands [176,177]. During ischemic stroke, the interruption of cerebral blood flow severely restricts the delivery of oxygen and glucose, both of which are essential for mitochondrial oxidative phosphorylation. As a result, ATP production is rapidly inhibited, leading to a critical energy deficit [178]. This energy failure disrupts numerous cellular processes, including membrane potential maintenance and ion homeostasis, ultimately promoting neuronal dysfunction and cell death [179].

Functional mitochondria transfer has been reported to improve cellular energy metabolism [124]. Following a hypoxic insult, ATP levels in PC12 cells decrease significantly compared to those in normoxic conditions. Functional mitochondria transfer enhances ATP production in hypoxic PC12 cells. When cocultured with MSCs, ATP content in hypoxic PC12 cells increases, and donor-derived mitochondria are visibly localized within recipient cells [124]. This improvement in energy metabolism is attributed to the functional restoration of damaged mitochondria. Numerous studies have demonstrated that functional mitochondria transfer elevates ΔΨm and reduces ROS levels in recipient cells [15,124,180]. Notably, after MSC administration in stroke models, the activities of key mitochondrial enzymes, such as CS, PDH, and α-KGDH, are enhanced in the recipient cells [124]. These enzymes are closely related to the TAC cycle and glycolysis, reflecting the restoration of the mitochondrial energy factory. Consistent with these findings, ρ^0^ PC12 cells with mtDNA depletion exhibit impaired oxidative phosphorylation and rely predominantly on anaerobic glycolysis, leading to elevated lactate accumulation in the culture medium [164]. Upon supplementation with MSCs, lactate levels in ρ^0^ PC12 cells return to near-normal values, reflecting a metabolic shift from glycolysis to aerobic respiration [164]. Collectively, these results confirm that functional mitochondria transfer supports the recovery of energy metabolism in recipient cells. Although intercellular functional mitochondria transfer occurs under physiological conditions, transferred mitochondria appear to become functionally active in recipient cells primarily under stress, suggesting that exogenous mitochondria are preferentially utilized when endogenous mitochondrial function is compromised [12]. Accumulated ROS and calcium overload in the post-ischemic microenvironment serve as key drivers of excessive mitochondrial fission. While functional mitochondria transfer provides antioxidative protection, persistent underlying pathological stimuli in recipient cells could counteract the pro-fusion effects conferred by transplantation, potentially even re-triggering fission dominance. Thus, the regulatory impact of transferred mitochondria on kinetic equilibrium is likely dynamic and contingent upon the restoration of microenvironmental homeostasis. Assessing the temporal dynamics of mitochondrial network stability following transplantation *in vivo* remains crucial.

#### 5.2.4 Apoptosis inhibition

Neuronal apoptosis in ischemic stroke proceeds through two pathways: the intrinsic and extrinsic apoptotic pathways [181,182]. The intrinsic pathway is mitochondria-dependent and involves intracellular stress signaling. Under ischemic conditions, mitochondria release cytochrome *c, a* key pro-apoptotic molecule. After being released to the cytoplasm, cytochrome *c* associates with apoptotic protease activating factor-1 (Apaf-1) and other cofactors to form the apoptosome, leading to the activation of caspase enzymes and initiation of the apoptotic cascade [182,183]. In contrast, the extrinsic pathway is initiated by the binding of specific ligands to death receptors on the cell membrane [183,184]. Inflammatory signals released during ischemia activate these receptors, further amplifying apoptotic signaling. Under ischemic conditions, the release of inflammatory mediators can activate death receptors on the cell surface, further amplifying apoptotic signaling. Studies have demonstrated that the proportion of apoptotic mitochondria is significantly reduced following the transfer of functional mitochondria into injured neurons [15,130,159,185]. In a mouse model of focal cerebral ischemia, direct injection of astrocyte-derived extracellular mitochondrial particles into the peri-infarct cortex, followed by flow cytometric analysis of neurons 24 h later, revealed increased expression of phosphorylated AKT, a cell survival-related signal, and the anti-apoptotic factor B-cell lymphoma-extra large (BCL-XL), along with reduced levels of activated caspase-3 [15]. Capobianco et al. [159]. exposed human SH-SY5Y cells to glucose- and serum-free medium under 0.2 % oxygen for 24 h to induce oxygen-glucose deprivation (OGD), resulting in marked activation of caspase-3/7 in the affected cells. However, when oxygen-glucose deprivation/reoxygenation (OGD/R) treated SH-SY5Y cells were cocultured with healthy human neural stem cells (hNSCs), caspase-3/7 activation was absent in cells that received functional mitochondria from hNSCs [159]. These findings indicate that functional mitochondria transfer may inhibit the intrinsic apoptotic pathway and exert a protective, anti-apoptotic effect. Following internalization, transferred mitochondria fuse with the damaged mitochondrial network of recipient cells, restoring mitochondrial function and preventing the release of pro-apoptotic factors. Preservation of mitochondrial integrity suppresses the activation of apoptotic cascades and promotes neuronal survival under ischemic stress. However, the efficiency of such 'prioritized utilization' within the complex pathophysiological context *in vivo* (e.g., BBB disruption, inflammatory infiltration, glial scar formation) and whether it suffices to reverse widespread energy crises requires evaluation through more stringent *in vivo* models.

#### 5.2.5 BBB integrity maintenance

As a selectively permeable interface, the BBB, formed primarily by brain endothelial cells, safeguards the brain from harmful substances while maintaining intracranial homeostasis. Astrocytes, endothelial cells, pericytes, and microglia jointly regulate blood-brain exchange and engage in intercellular communication, collaboratively generating survival signals essential for preserving BBB integrity and central nervous system homeostasis [[186], [187], [188], [189]]. Among these components, astrocytes contribute to structural support and functional regulation of endothelial cells through direct contact via end-foot processes [131,190,191]. In addition, astrocytes modulate BBB permeability by releasing various bioactive molecules, including vascular endothelial growth factor (VEGF), matrix metalloproteinases (MMPs), and nitric oxide (NO) [190,192]. Following ischemic brain injury, ischemic stress alters the expression of tight junction proteins in cerebral endothelial cells and activates astrocytes, thereby increasing BBB permeability [193,194]. As a result, macromolecules and inflammatory cells from the circulation gain access to the brain parenchyma, exacerbating cerebral edema and increasing the risk of hemorrhage [195,196]. Maintaining the viability and function of both endothelial cells and astrocytes is critical for preserving BBB integrity and overall neurological function. Pisani et al. demonstrated that astrocytes can acquire functional mitochondria from both pericytes and endothelial cells in coculture [130]. In subsequent experiments, astrocytes were subjected to OGD, followed by reoxygenation for 24 h in a complete medium. When cocultured with a defined number of healthy pericytes, astrocytes exhibited enhanced mitochondrial uptake, which effectively rescued them from OGD/R induced apoptosis [130]. Additional *in vitro* studies show that healthy cerebral endothelial cells can transfer functional mitochondria to hypoxia-injured endothelial cells, thereby preserving BBB integrity through a compensatory mechanism [131]. Endothelial progenitor cells (EPCs) also possess the capacity to secrete extracellular vesicles [197]. When mitochondrial particles derived from EPCs were introduced into injured endothelial cells, increases in mitochondrial protein translocase of the outer mitochondrial membrane 40 (TOM40) expression, mtDNA copy number, and intracellular ATP levels were observed, accompanied by a reduction in BBB permeability [197]. These findings suggest that EPCs support the bioenergetic demands of damaged endothelial cells and contribute to the maintenance of BBB integrity, in part through functional mitochondria transfer at least. The protective mechanism may involve the restoration of respiratory chain function and ATP synthase activity via mtDNA supplementation, as well as the upregulation of fibroblast growth factor-2 (FGF-2), which exerts direct protective effects on endothelial cells [[197], [198], [199]].

## 6 Targeting therapeutic strategies

Neuronal injury is a critical pathological process of ischemic stroke. As previously outlined, suppressing the release of damaged mitochondria and their associated components and promoting the transfer of functional mitochondria are essential for reducing neuronal injury. In addition, mitochondrial transplantation has emerged as a promising therapeutic approach, improving neurological deficits following ischemic injury. Based on these mechanistic insights, therapeutic strategies are categorized into three principal modalities.

### 6.1 Inhibiting the release of mitochondrial fragments

Under ischemic-hypoxic conditions, mitochondrial damage frequently exceeds the compensatory capacity of mitophagy, resulting in increased mitochondrial membrane permeability and the subsequent release of dysfunctional mitochondrial fragments. This pathological cascade activates inflammatory signaling and promotes neuronal cell death, aggravating ischemic stroke progression and worsening clinical outcomes. Table 1 outlines the neuroprotective effects of natural compounds against CI/RI through the regulation of two mechanisms: mitophagy and mPTP.

**Table 1:** **Research advances of mitochondrial transmission in ischemic stroke.** PINK1/Parkin, PTEN-induced putative kinase 1/Parkin RBR E3 ubiquitin-protein ligase; Cyt *c*, Cytochrome *c*; mtDNA, Mitochondrial DNA; LC3B, Microtubule-associated protein 1A/1B-light chain 3B; VDAC1, Voltage-dependent anion channel 1; LC3-II, LC3-phosphatidylethanolamine conjugate; LC3-I, Cytosolic LC3; Bax/BCL2, BCL2-associated X protein/B-cell lymphoma 2; mPTP, Mitochondrial permeability transition pore; ERK, Extracellular signal-regulated kinase; Cyp D, Cyclophilin D; CD38, Cluster of Differentiation 38; cADPR, Cyclic ADP-ribose; Miro1 (RHOT1), Ras homolog gene family member T1; PGC1α, Peroxisome proliferator-activated receptor gamma coactivator 1-alpha; SIRT3, Sirtuin 3.

Interventions	Mechanisms	Function	Effect	Ref.
Resveratrol	• Activates PINK1/Parkin pathway.	• Increases ΔΨm; • Suppresses O_2_•^-^ production; • Decreases caspase-3.	• Maintains mitochondrial membrane integrity; • Inhibits Cyt *c* and mtDNA release.	[200]
Curcumin	• Promotes LC3B-VDAC1 co-localization; • Increases LC3-II/LC3-I ratio.	• Increases MMP and ATP.	• Inhibits ROS production; • Maintains mitochondrial integrity.	[201]
Garciesculenxanthone B	• Activates Pink1/Parkin pathway.	• Decreases TOM20.	• Maintains mitochondrial membrane stability.	[202]
Dengzhan Xixin Injection (DX)	• Activates PINK1/Parkin.	• Reduces Bax, Bcl-2, and caspase-3.	• Reduces mitochondria-mediated apoptosis.	[203]
Cyclosporin A (CsA)	• Inhibits mPTP opening.	• Decreases caspase-3.	• Maintains mitochondrial morphological integrity; • Reduces mitochondria-mediated apoptosis.	[204]
Gallic acid	• Inhibits mPTP opening; • Inhibits ERK/CypD pathway.	• Decreases caspase-3.	• Reduces mitochondrial membrane permeability; • Decreases Cyt *c* release; • Reduces mitochondria-mediated apoptosis.	[205]
Ginsenoside Rb1	• Activates CD38/cADPR/Ca^2+^ pathway.	• Decreases CFAP; • Increases GSH and NADPH.	• Suppress astrocyte activation; • Inhibits ROS production; • Accelerates astrocyte-neuron functional mitochondria transfer.	[206]
Chrysophanol (CHR)	• Upregulates of Miro1 expression	• Increases MMP and ATP.	• Accelerates functional mitochondria transfer from astrocytes to neurons.	[207]
Melatonin	• Upregulates the expression PGC-1α and SIRT3	• Stabilizes ETC complexes; • Activates Nrf2 nuclear translocation; • Decreases LC3B-II/I and Beclin-1.	• Inhibits ROS production; • Increases astrocyte-neuron functional; • Promotes functional mitochondria transfer; • Suppresses excessive mitophagy.	[208,209]
Hyperthermia (33 °C)	/	• Increases MMP and ATP.	• Facilitates astrocyte-neuron functional mitochondria transfer.	[210]
Pretreatment with hyperbaric therapy	/	• Increases neuronal viability.	• Boost astrocyte -neuron functional mitochondria transfer.	[211]

Resveratrol mitigates OGD/R induced neuronal injury by stabilizing MMP, suppressing mitochondrial O_2_•^-^ production, and inhibiting caspase-3 activation. The protective effects rely on the activation of PINK1/Parkin-mediated mitophagy, as pharmacological inhibition of mitophagy significantly attenuates its efficacy [200]. Similarly, curcumin promotes colocalization of LC3B with VDAC1, enhances MMP and intracellular ATP levels, and activates the nuclear factor erythroid 2-related factor 2 (Nrf2)/antioxidant response element (ARE) pathway, suggesting a synergistic interaction between antioxidant defense and autophagic flux [201]. Garcinia-derived xanthone (GeB) stabilizes PINK1 and promotes Parkin translocation to depolarized mitochondria, thereby reducing infarct volume and suppressing neuronal apoptosis in a middle cerebral artery occlusion (MCAO) model [202]. Additionally, Dengzhan Xixin Injection (DX) and its active constituents, as predicted by molecular docking, target autophagy-related proteins such as LC3 and p62. These compounds increase LC3-Phosphatidylethanolamine Conjugate and Cytosolic LC3 ratios, reduce p62 expression, and concurrently modulate mitochondrial apoptotic pathways, exhibiting multi-target neuroprotective activity [203].

Cyp D, a central regulator of mPTP, interacts with ANT to induce pore opening, leading to ΔΨm collapse and cytochrome *c* release. The pathological relevance of Cyp D is supported by a 62 % reduction in infarct volume and improved mitochondrial calcium buffering capacity in Cyp D knockout mice [204]. Pharmacological inhibition of Cyp D includes agents such as cyclosporine A (CsA), which directly binds to Cyp D to prevent mPTP opening [204], and gallic acid, which reduces mPTP sensitivity via extracellular signal regulated kinase (ERK) dependent downregulation of Cyp D expression [205]. Both strategies attenuate mitochondrial swelling and suppress caspase cascade activation. Notably, the neuroprotective effects of gallic acid are abolished by ERK pathway inhibition or Cyp D overexpression, highlighting its dependence on the ERK-Cyp D signaling axis [205].

### 6.2 Intervention of functional mitochondria transfer

Neural energy demands are supported by an extensive mitochondrial network, which is dynamically redistributed through intercellular functional mitochondria transfer to maintain metabolic homeostasis and prevent stress-induced apoptosis. Endogenous transfer of healthy mitochondria represents a key compensatory mechanism for restoring mitochondrial function in injured or energy-deprived cells. Table 1 organizes interventions that promote functional mitochondrial transfer.

Ginsenoside Rb1 enhances neuroprotection following ischemic stroke by promoting the transfer of functional mitochondria from astrocytes to neurons [206]. Through reversible inhibition of mitochondrial complex I, Rb1 stabilizes ΔΨm and prevents fragmentation in astrocytes, thereby preserving mitochondrial integrity. This protective effect facilitates the release of functional mitochondria into the extracellular environment, as evidenced by increased mitochondrial signal and ATP levels in astrocyte-conditioned medium [206]. The transfer process is partially mediated by CD38, as silencing CD38 expression significantly diminishes Rb1's ability to rescue neuronal damage. Concurrently, Rb1 suppresses reverse electron transport (RET)-derived ROS production in astrocytes, which alleviates oxidative stress and calcium overload—key drivers of astrocyte activation. By coupling functional mitochondria transfer with ROS reduction, Rb1 ensures neuronal survival and functional recovery in ischemic conditions [206]. Chrysin (CHR) promotes functional mitochondria transfer from astrocytes to neurons, improving mitochondrial morphology and function in recipient neurons [207]. *In vivo* experiments have demonstrated that CHR can reduce CHR administration, reduce neurological deficit scores and infarct volume, enhance cellular morphology in the ischemic penumbra, and upregulate Miro1 expression, a key regulator of functional mitochondria transfer [207]. Melatonin treatment significantly increased the formation of TNTs between normal Neuro-2a (N2a) cells and mtDNA-deficient cells, which exhibited a higher frequency of mitochondrial fusion. After receiving functional mitochondria, recipient cells can stabilize electron transport chain complexes, improve the cellular ROS environment, and inhibit apoptosis. Furthermore, melatonin inhibits glial fibrillary acidic protein (GFAP) expression to reduce astrocyte activation, suppresses glial scar formation, and protects myelin structure; it also inhibits Aquaporin-4 (AQP4) expression to alleviate cerebral edema. Melatonin enhances the nuclear translocation of the redox-sensitive transcription factor Nrf2 in the oxidative stress microenvironment, which is crucial for balancing mitochondrial ROS production and maintaining cellular redox homeostasis [208,209]. Beyond pharmacological approaches, certain physical therapies have been shown to enhance functional mitochondria transfer efficiency. Mild hypothermia (approximately 33 °C) promotes spontaneous release of functional mitochondria from astrocytes into the extracellular environment, facilitating uptake by neurons and resulting in increased ATP levels, ΔΨm, and overall cellular viability [210]. Similarly, hyperbaric oxygen therapy enhances functional mitochondria transfer from astrocytes to neurons, contributing to improved neuronal survival under ischemic conditions [211].

### 6.3 Mitochondrial transplantation

Mitochondria transplantation is a procedure in which mitochondria are isolated from a cellular source and delivered postnatally to an individual with the specific purpose of eliciting a therapeutic response [212]. Table 2 demonstrates the significant therapeutic potential of mitochondrial transplantation in models of ischemic stroke. Its mechanisms encompass enhancing mitochondrial function, attenuating oxidative stress, suppressing cell death, modulating immune responses, and promoting tissue repair [[213], [214], [215]].

**Table 2:** **Research progress in mitochondrial transplantation for ischemic stroke.** ATP, Adenosine triphosphate; Bax, BCL2-associated X protein; Bcl-2, B-cell lymphoma 2; Cyt *c*, Cytochrome *c*; FABP5, Fatty acid-binding protein 5; FABP7, Fatty acid-binding protein 7; GFAP, Glial fibrillary acidic protein; GSH-Px, Glutathione peroxidase; hUC-MSCs, Human umbilical cord mesenchymal stem cells; Iba-1, Ionized calcium-binding adapter molecule 1; N2a cells, Neuro-2a cells; NLRP3, NLR family pyrin domain containing 3; GSDMD, Gasdermin D; OPC, Oligodendrocyte precursor cell; ROS, Reactive oxygen species; SD rats, Sprague-Dawley rats; SOD, Superoxide dismutase.

Animal species	Mitochondrial sources	Injection sites and methods	Function	Therapeutic outcomes	Ref.
Male SD rats	SD rat pectoralis major	Lateral ventricle injection	• Increases SOD and GSH-Px increased; • Decreases caspase 3 and Cyt *c*; • Reduces GFAP.	• Inhibits oxidative stress; • Inhibits neuronal apoptosis; • Reduces astrocyte activation.	[216]
MaleC57BL/6 mice	C57BL/6 mouse liver	Tail vein injection/Intracerebral injection	• Inhibits NLRP3, GSDMD expression	• Inhibits microglial pyroptosis; • Promotes neurogenesis	[221]
MaleC57BL/6 mice	C57BL/6 mouse liver	Intracerebral injection	• Reduces caspase-3; • Upregulates FABP5, FABP7.	• Promotes OPC survival and proliferation; • Promotes myelination.	[223]
MaleC57BL/6 mice	E17 mouse placenta	Intravenous injection	•Increases ATP.	• Reduces infarct area.	[214]
Male SD rats	Hamster kidney fibroblasts	Intracerebral Injection/Femoral artery inject	• Increases ATP; • Increases Iba-1 and GFAP.	• Reduces apoptosis; • Reduces glial cell hyperactivity.	[215]
Wistar rats	hUC-MSCs	Intracerebroventricular injection	• Decreases Iba-1.	• Reduces apoptosis; • Reduces glial cell hyperactivity.	[220]
SD rats	N2a cells	Internal carotid artery injection	• Reduces Bax, Bcl-2, and caspase-3.	• Inhibits ROS production; • Reduces apoptosis.	[217]

Specifically, studies confirm that mitochondrial transplantation effectively enhances systemic antioxidant capacity. For instance, transplantation of autologous mitochondria isolated from rat skeletal muscle (e.g., pectoralis major) into the lateral ventricle significantly increased neuronal levels of key antioxidant enzymes, including SOD and GSH-Px, by approximately 3-fold compared to controls [216]. Furthermore, transplantation of mitochondria derived from N2a cells markedly reduced intracellular ROS levels in neurons [217]. Similarly, transplantation of extracellular vesicles from mouse endothelial cells into mouse brain endothelial cells exerted significant *anti*-ROS effects [218]. These antioxidative effects coincided with effective inhibition of apoptotic pathways, particularly the modulation of key apoptotic factors such as the Bax/Bcl-2 ratio, caspase-3, and cytochrome *c* [216,218]. Mitochondrial transplantation potentially modulates immune cell activation, differentiation, and survival through mechanisms involving the reduction of oxidative stress and regulation of cell death [219]. Supporting this, intracerebroventricular injection of healthy mitochondria derived from human umbilical cord mesenchymal stem cells (hUC-MSCs) into ischemic rat models resulted in a significant decrease in the number of Iba-1 positive microglia, indicating suppression of microglial overactivation [220]. Notably, studies revealed that the DAMP S100A9, which is significantly upregulated in ischemic stroke, promotes the engulfment of exogenous functional mitochondria by microglia [221]. This process subsequently reduces microglial pyroptosis and promotes their proliferation and polarization toward an anti-inflammatory phenotype [221]. Furthermore, the study revealed that functional mitochondria transfer also exerts a significant influence on the peripheral immune system. Cerebral ischemia induces glycolysis in peripheral CD4^+^ T cells, leading to a Th17/Treg imbalance. Umbilical cord mesenchymal stem cells can mitigate this imbalance by transferring mitochondria to T cells, thereby reducing the production of inflammatory cytokines [222]. Addressing the therapeutic challenge of axonal degeneration and demyelination post-ischemia, mitochondrial transplantation also exhibits beneficial effects. Myelin regeneration is highly dependent on the energy supply to oligodendrocyte progenitor cells (OPCs). Research indicates that improving mitochondrial function in OPCs via transplantation enhances their survival and proliferation in ischemic brain tissue, evidenced by increased numbers of proliferating OPCs and decreased apoptotic OPCs. This process is further associated with elevated myelin basic protein (MBP) levels, restoration of normal myelin morphology, improved Olig2 expression and lipid synthesis signaling, and metabolic reprogramming [223]. Concurrently, mitochondrial transplantation reduces GFAP expression, thereby mitigating glial scar formation (which typically impedes neural regeneration) and inducing neurogenesis in the ischemic penumbra [220].

Collectively, these effects, including attenuated oxidative stress, suppressed apoptosis and pyroptosis, modulated immune responses, promoted myelin repair, and induced neurogenesis, converge to form a complex mechanistic network underpinning mitochondrial transplantation facilitated brain tissue repair. Ultimately, this therapy effectively reduces cerebral infarct volume and improves neurobehavioral function in ischemic stroke models.

## 7 Conclusion and perspectives

Dysfunctional and functional mitochondria exert a critical influence on the pathogenesis, progression, and clinical outcomes of ischemic stroke. The present review systematically delineates the mechanistic framework and functional consequences of dysfunctional mitochondrial fragments release versus functional mitochondria transfer, clarifying their opposing roles in regulating neuroinflammation and mediating cellular salvage. These insights offer new conceptual foundations for understanding mitochondrial pathophysiology in ischemic stroke therapy.

Under physiological conditions, damaged mitochondria are selectively eliminated through mitophagy to preserve mitochondrial quality control. When mitochondrial injury surpasses the capacity of mitophagy, mitochondrial components escape into the cytoplasm via mechanisms such as mPTP opening, BAX/BAK oligomerization, and GSDMD-mediated pore formation. These fragments subsequently activate inflammatory pathways through cGAS-STING signaling, the NLRP3 and AIM2 inflammasomes, and Toll-like receptors, culminating in regulated cell death. Within the neurovascular unit, these responses manifest as microglial M1 polarization, A1 astrocyte transformation, BBB disruption, neuronal apoptosis, and enhanced infiltration of peripheral immune cells. In contrast, functional mitochondria transfer via TNTs and EVs serves as an intrinsic neuroprotective mechanism. During ischemic injury, neurons transfer functional mitochondria to astrocytes through CD38 or LRP1-mediated EVs trafficking for lysosomal degradation, thereby supporting mitochondrial quality homeostasis. Stem cell-derived functional mitochondria transfer through TNTs promotes neuronal recovery, with Miro1 identified as a key regulator of mitochondrial trafficking. TNTs mediated mitochondrial exchange among pericytes, endothelial cells, and astrocytes also play a crucial role in maintaining BBB integrity. EVs-mediated functional mitochondria transfer typically involves uptake through endocytosis or macropinocytosis, followed by fusion with host mitochondrial networks via MFN1/2 and OPA1-dependent processes. However, emerging studies suggest that exogenous mitochondria may not always integrate directly with endogenous mitochondria but instead activate recipient cell responses via the PINK1-Parkin mitophagy pathway [161]. Building upon these mechanistic insights, several therapeutic strategies have been proposed, including inhibition of dysfunctional mitochondrial fragments release, enhancement of functional mitochondria transfer, and mitochondrial transplantation.

Despite substantial progress in elucidating the roles of dysfunctional mitochondrial fragments release and functional mitochondria transfer in cerebral pathology, several critical questions remain unresolved. First, while mitochondrial transfer shows potential in mitigating cellular oxidative stress, current research faces two limitations: the exact mechanism remains obscure, and the specific components and origins of the reduced oxidative stress products have not been sufficiently traced or studied. Second, although dysfunctional mitochondrial fragments trigger inflammatory responses, the dual role of inflammation in disease progression requires further clarification, particularly regarding the threshold of mitochondrial damage necessary to initiate detrimental outcomes. Third, given that both dysfunctional fragments and functional mitochondria are transported via EVs, the presence of residual fragments within mitochondria bearing EVs and the molecular mechanisms governing cargo selectivity remain to be defined. Fourth, the mechanisms by which functional mitochondria evade lysosomal degradation to sustain energy metabolism in ischemic neurons have not yet been fully elucidated. Clarifying these processes may enhance the efficacy of exogenous mitochondrial delivery and further inform research in cellular bioenergetics. Fifth, while functional mitochondria transfer from microglia to neurons via TNTs has been observed in neurodegenerative models, the presence and functional relevance of such interactions in ischemic stroke remain unverified [224]. Sixth, emerging evidence indicates that macrophage-mediated functional mitochondria transfer to cardiomyocytes induces iron overload, challenging the assumption that functional mitochondria transfer is universally beneficial [225], necessitating systematic evaluation of context-dependent outcomes. Seventh, as semi-autonomous organelles, mitochondria may influence recipient cell genetics through mtDNA transfer, requiring rigorous investigation into the long-term consequences of mtDNA integration into host genomes. Eighth, the therapeutic approach of directly adding mitochondria or donor cells to a culture dish *in vitro* is difficult to replicate *in vivo*. How to efficiently, specifically, and safely deliver functional mitochondria or their donor cells to specific cell types (e.g., dying neurons) within ischemic brain tissue? This requires overcoming multiple barriers, including the BBB, systemic clearance, capillary occlusion, and cell-specific uptake. The use of engineered vesicles (e.g., targeted EVs loaded with mitochondria) or novel nanocarriers represents an active research direction, though their efficiency, targeting precision, and intracerebral distribution require further optimization. Ninth, *in vitro* studies struggle to determine the minimum effective quantity of mitochondria or donor cells needed for significant neuroprotection *in vivo*, as well as the optimal therapeutic time window (Is efficacy limited to the acute phase? Does the subacute phase retain therapeutic value?). Tenth, *in vitro* experiments typically assess short-term effects (hours to days). Critical long-term safety concerns—such as the stability of transferred mitochondria (especially exogenous ones) within recipient cells, potential immune reactions (e.g., allogeneic mtDNA acting as DAMPs), impacts on mtDNA heteroplasmy in recipient cells, and tumorigenic risks (particularly in stem cell-mediated transfer)—remain largely unexplored in *in vivo* contexts. In summary, despite numerous encouraging *in vitro* studies (as reviewed in Section 5.2 of this article) revealing the multifaceted protective mechanisms of functional mitochondria transfer against ischemic stroke injury, we must critically acknowledge the descriptive nature of these findings and the substantial gaps in their *in vivo* validation and clinical translation. The core limitations of current research lie in the chasm between the oversimplified *in vitro* models and the extreme complexity of *in vivo* pathophysiological environments, coupled with technical challenges in dynamically monitoring transfer processes and evaluating functional relevance *in vivo*. Addressing these unresolved questions will refine therapeutic strategies and deepen our understanding of mitochondrial multifunctionality. Ultimately, elucidating the mechanisms governing dysfunctional mitochondrial release and functional intercellular transfer holds significant promise for ischemic stroke therapy.

## CRediT authorship contribution statement

**Lan Yang:** Writing – original draft, Visualization, Software, Data curation. **Xuan Wei:** Visualization. **Zi Liao:** Visualization. **Bei Chen:** Visualization. **Guang Zeng:** Supervision. **Zhigang Mei:** Supervision, Funding acquisition.

## Funding information

This work was supported by the 10.13039/501100001809National Natural Science Foundation of China, [Grant/Award Number: 82174167]; the Project of 10.13039/501100004735Natural Science Foundation of Hunan Province, [Grant/Award Number: 2023JJ30464], [Grant/Award Number: 2025JJ90001]; Furong Scholars Program of Hunan Province, [Grant/Award Number: 2024]; the Young Qihuang Scholar Support Project of National Administration of Traditional Chinese Medicine, [Grant/Award Number: 2022]; Postgraduate scientific Research innovation Project of 10.13039/501100014978Hunan University of Chinese Medicine, [Grant/Award Number: 2024CX102].

## Declaration of competing interest

The authors declare no conflict of interest in the manuscript.

## Figures and Tables

**Table:** 

AIM2	Absent in melanoma 2
AML	Acute myeloid leukemia
ANT	Adenine nucleotide translocase
Apaf-1	Apoptotic protease activating factor-1
AQP4	Aquaporin-4
ARE	Antioxidant response element
ARF1	ADP-ribosylation factor 1
ATP	Adenosine triphosphate
α-KGDH	α-Ketoglutarate dehydrogenase
BAK1	BCL2 antagonist/killer 1
BAX	BCL2-associated X protein
BBB	Blood-brain barrier
BCL-XL	B-cell lymphoma-extra large
BM MSCs	Bone marrow mesenchymal stem cells
βCaMKII	Calcium/calmodulin-dependent protein kinase II beta
cADPR	Cyclic ADP-ribose
cGAMP	Cyclic GMP-AMP
cGAS	Cyclic GMP-AMP synthase
CHR	Chrysin
CI/RI	Cerebral ischemia/reperfusion
CL	Cardiolipin
CNS	Central nervous system
CS	Citrate synthase
Cyp D	Cyclophilin D
Cyt *c*	Cytochrome *c*
CsA	Cyclosporine A
DAMPs	Damage-associated molecular patterns
DRP1	Dynamin-related protein 1
dsDNA	Double-stranded DNA
DX	Dengzhan xixin injection
ECAR	Extracellular acidification rate
EGFR	Epidermal growth factor receptor
EPCs	Endothelial progenitor cells
ER	Endoplasmic reticulum
ERK	Extracellular signal-regulated kinase
ETC	Electron transport chain
EVs	Extracellular vesicles
FABP5	Fatty acid-binding protein 5
FABP7	Fatty acid-binding protein 7
Fis1	Fission protein 1
GeB	Garcinia-derived xanthone
GFAP	Glial fibrillary acidic protein
GTP	Guanosine triphosphate
Golgi	Golgi apparatus
GSDMD	Gasdermin D
GSDME	Gasdermin E
GSH	glutathione
GSH-Px	Glutathione peroxidase
GSSG	Glutathione disulfide
HO-1	Heme oxygenase-1
hNSCs	Human neural stem cells
hUC-MSCs	Human umbilical cord mesenchymal stem cells
H_2_O_2_	hydrogen peroxide
IMM	Inner mitochondrial membrane
IKK	IκB kinase
IL-1β	Interleukin-1 beta
IL-6	Interleukin-6
IMS	Intermembrane space
IRAKs	Interleukin-1 receptor-associated kinases
IRF3	Interferon regulatory factor 3
ISCs	Iron-sulfur clusters
LC3	Microtubule-associated protein 1A/1B light chain 3
LC3-I	Cytosolic LC3
LC3-II	LC3-phosphatidylethanolamine conjugate
LRP1	Lipoprotein receptor-related protein 1
MAPK	Mitogen-activated protein kinase
MBP	Myelin basic protein
MCAO	Middle cerebral artery occlusion
MDA	Malondialdehyde
MFN1	Mitofusin 1
MFN2	Mitofusin 2
Miro1	Mitochondrial Rho GTPase 1
MMPs	Matrix metalloproteinases
mPTP	Mitochondrial permeability transition pore
MQC	Mitochondrial quality control
mTOR	Mechanistic target of rapamycin
MSCs	Mesenchymal stem cells
mtDNA	Mitochondrial DNA
NAC	N-acetylcysteine
N2a	Neuro-2a
NF-κB	Nuclear factor kappa-light-chain-enhancer of activated B cells
NLRP3	NLR family pyrin domain containing 3
NMDA	N-Methyl-d-aspartate
NO	Nitric oxide
NOX2	NADPH oxidase 2
Nrf2	Nuclear factor erythroid 2-related factor 2
NVU	Neurovascular unit
O-GlcNAcylation	O-linked β-N-acetylglucosaminylation
OCR	Oxygen consumption rate
OGD	Oxygen-glucose deprivation
OGD/R	Oxygen-glucose deprivation/reoxygenation
OMM	Outer mitochondrial membrane
OPA1	Optic atrophy 1
OPCs	Oligodendrocyte progenitor cells
OXPHOS	Oxidative phosphorylation
ox-mtDNA	Oxidized mitochondrial DNA
OX	xanthine oxidase
P2X7	P2X purinoceptor 7
Parkin	Parkin RBR E3 ubiquitin-protein ligase
PDH	Pyruvate dehydrogenase
PGC-1α	Peroxisome proliferator-activated receptor gamma coactivator 1-alpha
PI3K	Phosphoinositide 3-kinase
PINK1	PTEN-induced putative kinase 1
PPAR	Peroxisome proliferator-activated receptor
PRRs	Pattern recognition receptors
RalA	RAS-like protein A
RHOT1	Rho GTPase 1
ROS	Reactive oxygen species
RyR	Ryanodine receptor
S100A4	S100 calcium-binding protein A4
SIRT-1	Sirtuin 1
SLC35B4	Solute carrier family 35 member B4
STING	Stimulator of interferon genes
SOD	Superoxide dismutase
TBK1	TANK-binding kinase 1
TCA cycle	Tricarboxylic acid cycle
TFAM	Mitochondrial transcription factor A
TLRs	Toll-like receptors
TNF-α	Tumor necrosis factor-alpha
TOM20	Translocase of the outer mitochondrial membrane 20
TOM40	Translocase of the outer mitochondrial membrane 40
TRAF6	TNF receptor-associated factor 6
TRAK1/2	Trafficking kinesin proteins 1 and 2
VDACs	Voltage-dependent anion channels
VEGF	Vascular endothelial growth factor
Wnt5a	Wingless-type mmtv integration site family, member 5a
XDH	xanthine dehydrogenase
ΔΨm	Mitochondrial membrane potential
•OH	highly oxidizing hydroxyl radical
O_2_•^-^	superoxide anion

## Data Availability

No data was used for the research described in the article.
